# Long-term outcomes of induction chemotherapy followed by concurrent chemoradiotherapy and adjuvant chemotherapy for locoregionally advanced nasopharyngeal carcinoma: a retrospective study

**DOI:** 10.3389/fonc.2024.1475176

**Published:** 2024-11-27

**Authors:** Xiaoyan Zhao, Ling Tian, Yun Chen, Qing Yang, Tao Xie, Modong Chen, Jinhui Rao, Meng Yang, Ning Huang, Yanxin Ren

**Affiliations:** ^1^ Department of Head and Neck Surgery, Third Affiliated Hospital of Kunming Medical University, Kunming, Yunnan, China; ^2^ Department of Pathology, Third Affiliated Hospital of Kunming Medical University, Kunming, Yunnan, China; ^3^ Department of Pharmacology, School of Basic Medicine, Kunming Medical University, Kunming, Yunnan, China

**Keywords:** nasopharyngeal carcinoma, adjuvant chemotherapy (AC), induction chemotherapy (IC), overall survival (OS), progression-free survival (PFS)

## Abstract

**Background:**

Nasopharyngeal carcinoma (NPC) is a prevalent form of head and neck cancer, particularly in specific regions with a higher incidence. The optimal treatment strategy for locally advanced NPC (stage III and IVA, LA-NPC) involves various combinations of induction chemotherapy (IC), concurrent chemoradiotherapy (CCRT), and adjuvant chemotherapy (AC), each with distinct advantages. This one institutional study aims to retrospectively analysis the efficacy and clinical outcomes of IC with CCRT (IC+CCRT), CCRT with AC (CCRT+AC), and the comprehensive approach of IC followed by CCRT and subsequently AC (IC+CCRT+AC) in the management of LA-NPC.

**Materials and methods:**

A total of 352 LA-NPC patients were included: 173 accepted IC+CCRT, 60 received CCRT+AC, and 119 underwent IC+CCRT+AC. The primary endpoints including overall survival (OS) and progression-free survival (PFS), were assessed using the Kaplan-Meier method and log-rank test.

**Results:**

The median follow-up was 61.2 months (1-216 months). There was no significant difference in 5-year OS and PFS between IC group and no IC group, extending the observation time to 90 months, the OS and PFS were significantly better in IC group than no IC group (OS: 76% vs. 70%,P<0.05; PFS: 76% vs. 71%, P<0.05). Patients with 1, 2, or 3 cycles of IC had higher 5-year OS and PFS than those with more than 3 cycles (1-4 cycles IC OS: 89% vs. 87% vs. 88% vs. 79%, P<0.05; 1-4 cycles IC PFS: 87% vs. 85% vs. 85% vs. 70%, P<0.05). NP regimen demonstrated higher OS and PFS than TP, PF, and TPF regimens (OS: 95% vs. 82% vs. 85% vs. 71%, P<0.05; PFS: 93% vs. 83% vs. 81% vs. 80%, P<0.05). The 5-year OS and PFS were significantly better in AC group than no AC group (OS: 82% vs. 72%, P<0.05; PFS: 81% vs. 69%, P<0.05). In the AC group, there was no differential effect of chemotherapy cycles and chemotherapy regimens on patients’ OS and PFS. In the ThNh group, patients receiving IC+CCRT+AC had higher OS and PFS compared to those receiving IC+CCRT, with no significant difference in the rest (OS: 85% VS 66% P<0.05; PFS: 78% VS 62%, P<0.05).

**Conclusion:**

CCRT combined with IC or AC could benefit LA-NPC patients. The IC+CCRT +AC regimen was most beneficial for NPC patients with later T and N stages.

## 1 Introduction

Nasopharyngeal carcinoma (NPC) is one of the most common forms of head and neck cancer. GLOBOCAN cancer estimated nearly 130,000 fresh NPC cases in 2018, which is nearly 0.7% of worldwide cancer cases in 2018, with the highest incidence rates in regions in North Africa, Southeastern Asia, and South China. Geographic differences abound. In Asia, Vietnam has one of the highest NPC mortality rates, nearly 5 times the global rate. While China and the Philippines report NPC mortality rates 2 times higher than the global rate, South Korea, India, and Japan all report rates significantly lower. In the United States, most studies on NPC mortality have not disaggregated Asian subgroups to examine within-group variation. A 25-year study of NPC in Asians ending in 2009 found Asian Americans to have a 5.6 times higher age-adjusted mortality rate (AAMR) than non-Hispanic white (NHW), 6.7 times higher than Hispanic white (HW), and 3.3 times higher than blacks (1).

Around 70% of these cases were initially diagnosed as locoregionally advanced, necessitating an in-depth exploration of the disease’s clinical landscape, treatment modalities, and recent advancements in managing LA-NPC (2). LA-NPC is associated with a poor prognosis, characterized by elevated rates of locoregional recurrence and distant metastasis. Recognizing its clinical complexity, the National Comprehensive Cancer Network (NCCN) advocates a multimodal approach, combining radiotherapy and chemotherapy, as the standard of care for LA-NPC (3). A significant trial evaluating the efficacy and toxicity of TPF-based IC in LA-NPC revealed substantial improvements in 5-year OS, PFS, distant metastasis-free survival, and localized progression-free survival when compared to historical benchmarks (4). It is imperative to underscore patients dealing with LA-NPC, even when undergoing intensity-modulated radiotherapy, necessitate the concurrent administration of IC and AC (5). A meta-analysis demonstrated that IC+RT+AC showcases exceptional 5-year local recurrence control and distant failure-free survival rates, standing at 90.3% and 79.4%, respectively (6). Both IC and AC are employed to enhance locoregional control and can support early treatment for occult micrometastases or distant metastases. However, as for our current knowledge, determining the optimal combination “cocktail” remains a matter of controversy (7, 8). The object of this retrospective study is to contribute in shedding light on the optimal treatment modality for LA-NPC. We analyzed 352 diagnosed patients with NPC from the Third affiliated hospital of Kunming medical university. These clinical data represent a helpful treatment benchmark for treatment of LA-NPC.

## 2 Methods

### 2.1 Case eligibility

A retrospective review was conducted of NPC patients treated at the Third affiliated hospital of Kunming medical university between January 1, 2005, and December 31, 2010. All participants in this study met specific eligibility criteria, ensuring a standardized cohort: (a)Confirmed diagnosis of NPC through biopsy; (b)Absence of distant metastasis; (c)No history of prior radiotherapy to the neck;(d)Pathologically confirmed stage III-IVA NPC according to the 8th edition of the Union for International Cancer Control/American Joint Committee on Cancer (UICC/AJCC) staging system;(e)Treatment regimens involved IC + CCRT, CCRT + AC, or IC + CCRT + AC based on Intensity-Modulated Radiation Therapy (IMRT).

Patients were classified into three groups based on T and N staging: (1) T high and N low (ThNl), (2) T low and N high (TlNh), and (3) T high and N high (ThNh) (**Supplementary Table S1**). The medical follow-up included medical history with toxicity evaluation, physical examination and imaging studies, scheduled as follows: every week during RT, every 3 months for the first 2years, every 4 to 6 months for the next 3 years and then annually. Local control was defined as no signs of tumor progression on endoscopy, CT or MRI scans. OS was defined as the date of histologic diagnosis to the date of death from any cause or last visit. PFS was defined the date of histologic diagnosis to the date of treatment failure or death, whichever occurred first.

### 2.2 Radiotherapy

CCRT uses a cisplatin single-agent chemotherapy regimen (40mg/m^2^, weekly); radiotherapy uses IMRT, with a total dose of 66-70Gy, and a single radiation therapy dose of 1.8-2.2Gy once a day for 5 consecutive days per week, followed by a 2-day break. It usually takes about 6-7 weeks to complete the total radiotherapy.

### 2.3 Chemotherapy

A total of 352 patients underwent CCRT. Patients received 1 to 4 cycles of IC and 1 to 6 cycles of AC. The regimens of AC and IC were same and included the following: TP (docetaxel 75 mg/m^2^ and cisplatin 75 mg/m^2^ on the first day), DF (cisplatin 80 mg/m^2^ on the first day and 5-fluorouracil 800 mg/m^2^/day as a continuous 120 h infusion on days 1–5), NP (vinorelbine 25 mg/m^2^ on the first and eighth days while cisplatin 80 mg/m^2^ on the first day), TPF (docetaxel 75 mg/m^2^ and cisplatin 80 mg/m^2^ on the first day while 5-fluorouracil 600mg/m^2/^day as a continuous 120 h infusion on days 1–5), which were administered every 3 weeks as a cycle. Among them, 179 received AC, with TP regimen (21.79%), DF regimen (49.16%), NP regimen (25.14%), TPF regimen (2.79%) and other regimens (1.12%) being administered. Additionally, 173 patients underwent IC, with TP regimen (24.86%), DF regimen (42.20%), NP regimen (23.70%), TPF regimen (5.78%), and other regimens (3.46%) being administered (**Supplementary Table S2**).

### 2.4 Statistics

All statistical analyses were conducted using SPSS20.0 (The R Foundation for Statistical Computing, Vienna, Austria). OS, PFS were measured from the first day of initial therapy and calculated by the Kaplan-Meier method. Differences between groups were tested using the Wilcoxon rank sum test by the Breslow method, A P value <0.05 was considered statistically significant.

## 3 Results

### 3.1 Baseline patient characteristics

352 patients LA-NPC (stage III and IVA) were enrolled:173 cases belonged to the IC + CCRT group, 60 cases to the CCRT + AC group, and 119 cases to the IC + CCRT + AC group. The male-to-female ratio was 2.87:1. Patients in group ThNl, TlNh and ThNh were123, 124 and 105. The ratio of non-smokers to smokers was 2.81:1, and the ratio of non-drinkers to drinkers was 1.03:1. (**Supplementary Table S3**).

### 3.2 Survival

**Figure 1:**
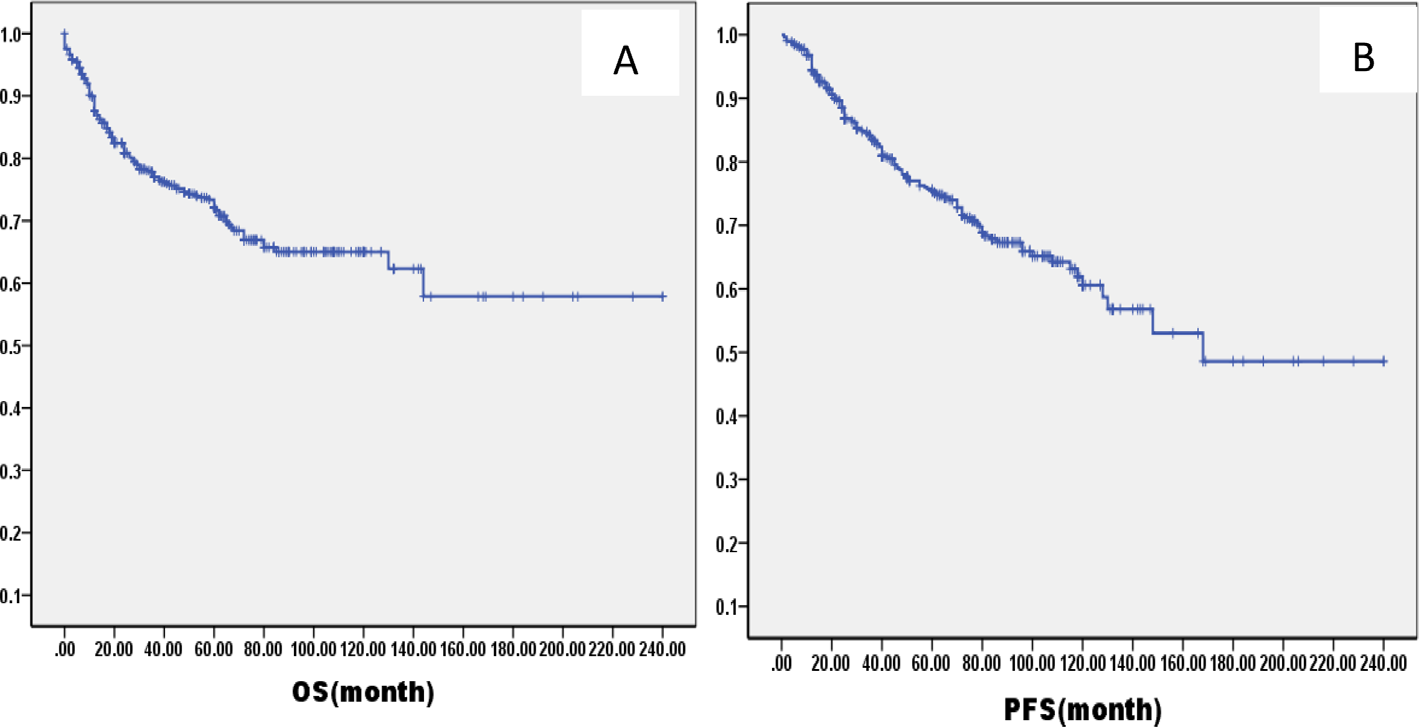
Kaplan–Meier curves of overall survival **(A)**, progression-free survival **(B)**, in all LA-NPC patients.

The median follow-up was 61.2 months (range: 1–216months) for all patients. The OS and PFS rates at 5 years were 79.0% (95% confidence interval [CI]: 66.9–89.2%) and 77.0% (95% CI: 64.3–85.1%), respectively (**Figure 1**). There were 60 cases of local recurrence, mainly concentrated in the nasopharynx and cervical lymph nodes, and 103 cases of distant metastasis, mainly in the lungs, liver, bone, and brain.

**Figure 2:**
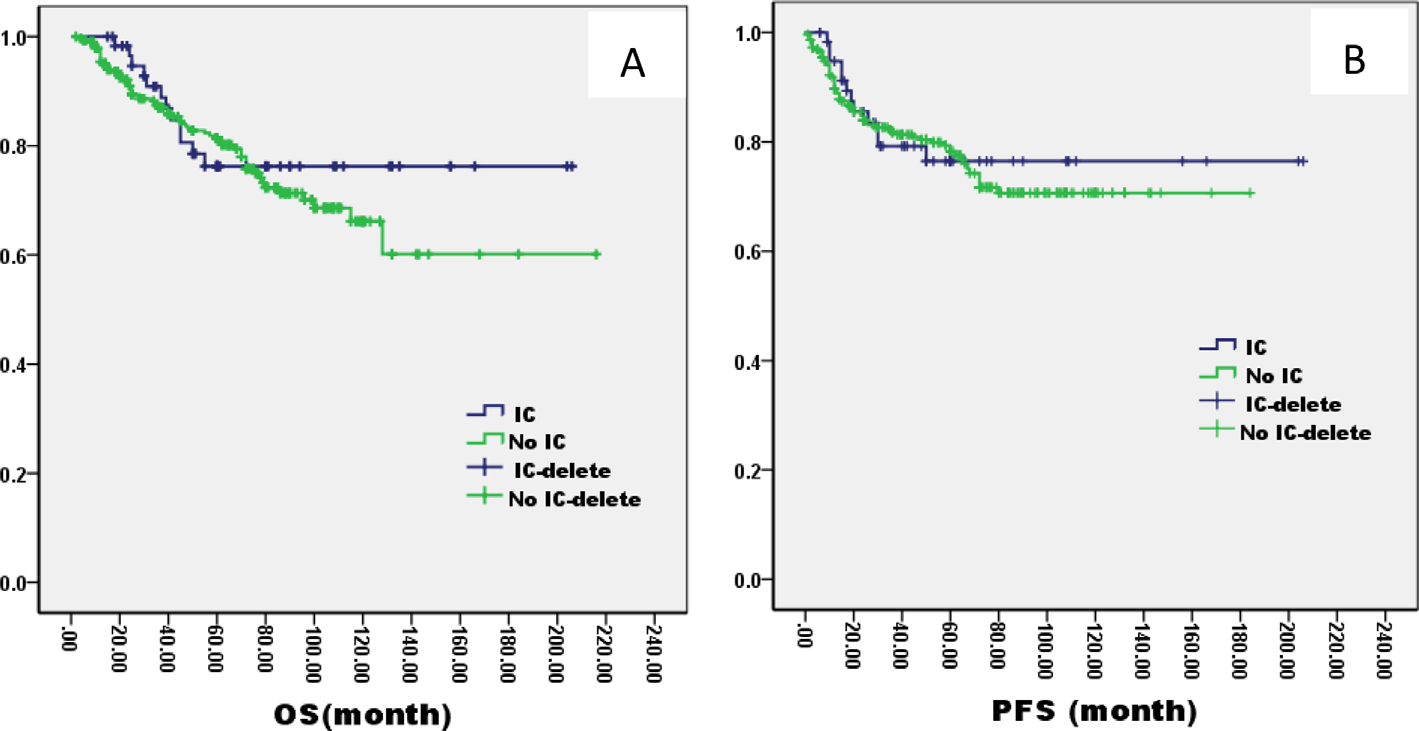
Kaplan–Meier curves of overall survival **(A)**, progression-free survival **(B)**, in LA-NPC patients treated with IC or not.

**Figure 3:**
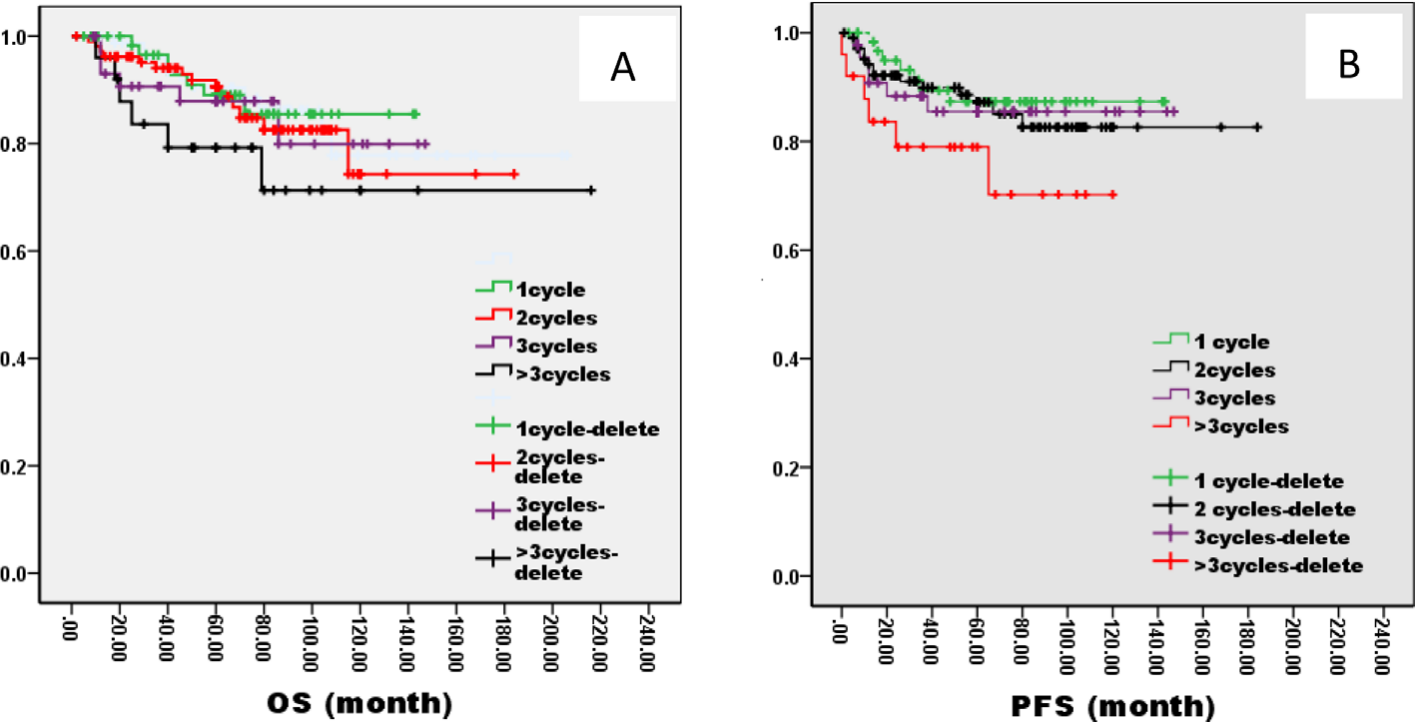
Kaplan–Meier curves of overall survival **(A)**, progression-free survival **(B)**, in LA-NPC patients treated with different IC cycles.

**Figure 4:**
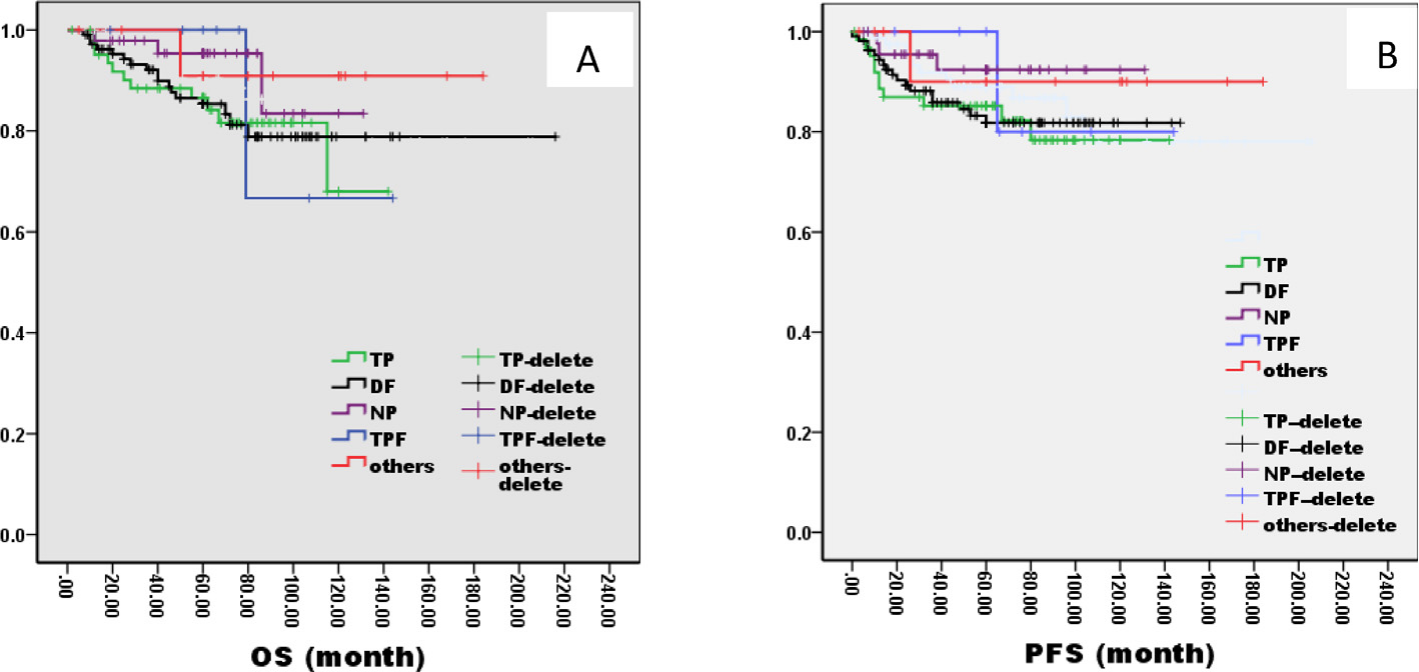
Kaplan–Meier curves of overall survival **(A)**, progression-free survival **(B)**, in LA-NPC patients treated with different chemotherapies.

There was no significant difference in 5-year OS and PFS between IC group and no IC group by the log-rank test in univariate analysis (OS: 76% VS 80%, P=1.121; PFS: 76% VS 75%, P=0.826, **Figure 2**). However, extending the observation time to 90 months, the OS and PFS were significantly better in IC group than no IC group by the log-rank test in univariate analysis (OS: 76% vs. 70%, P=0.02; PFS: 76% vs. 71%, P=0.03, **Figure 2**). In the IC group, we analysis the effect of different chemotherapy cycles and chemotherapy regimens on survival time. Cycles of chemotherapy had an effect on OS and PFS, which was higher in patients with 1, 2, or 3 cycles of IC than in patients with more than 3 cycles of IC (OS: 89% vs 87% vs 88% vs. 79%, P=0.03, 0.04, 0.04; PFS: 87% vs. 85% vs. 85% vs. 70%, P= 0.01, 0.02, 0.02, **Figure 3**). IC regimens include NP, TP, DF, TPF and others, and patients receiving NP regimens had higher OS and PFS than patients receiving TP, DF, and TPF regimens (OS: 95% vs. 82% vs. 85 vs. 71%, P= 0.015, 0.006, 0.01; PFS: 93% vs. 83% vs. 81% vs. 80%, P= 0.025, 0.04, 0.031, **Figure 4**).

**Figure 5:**
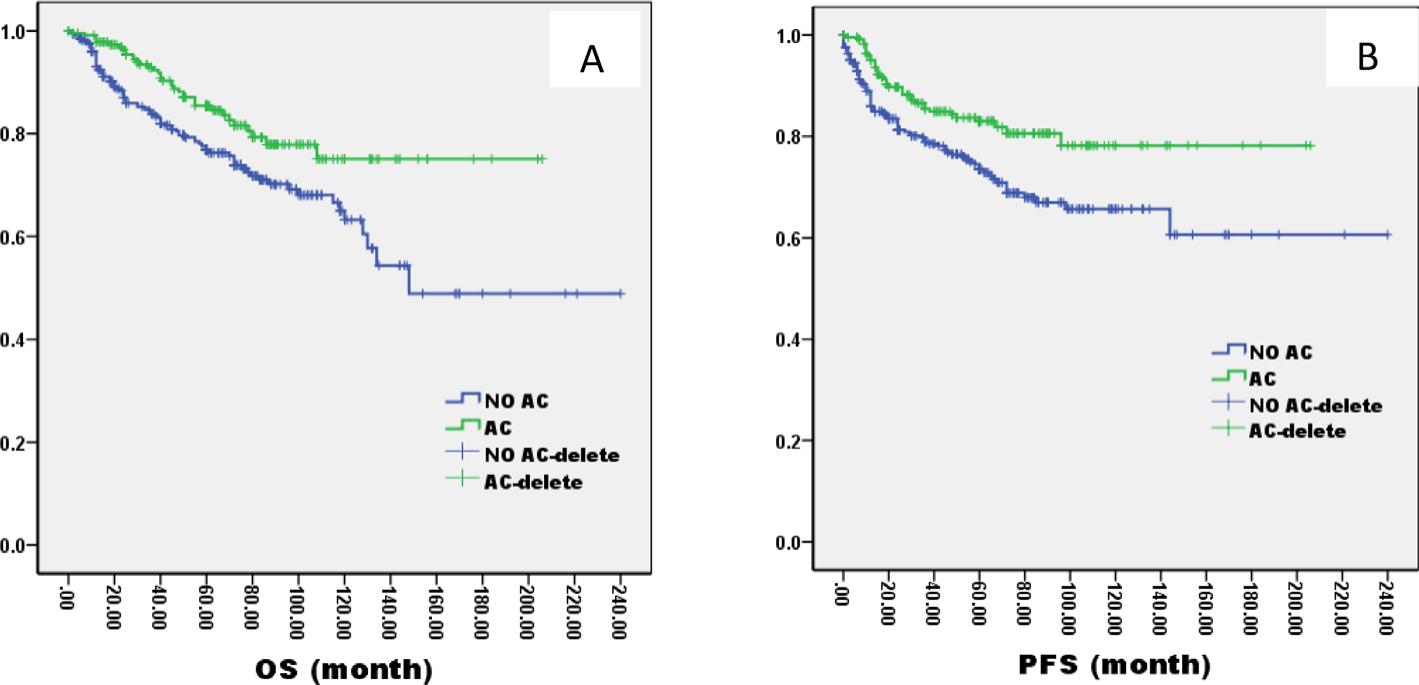
Kaplan–Meier curves of overall survival **(A)**, progression-free survival **(B)**, in LA-NPC patients treated with AC or not.

**Figure 6:**
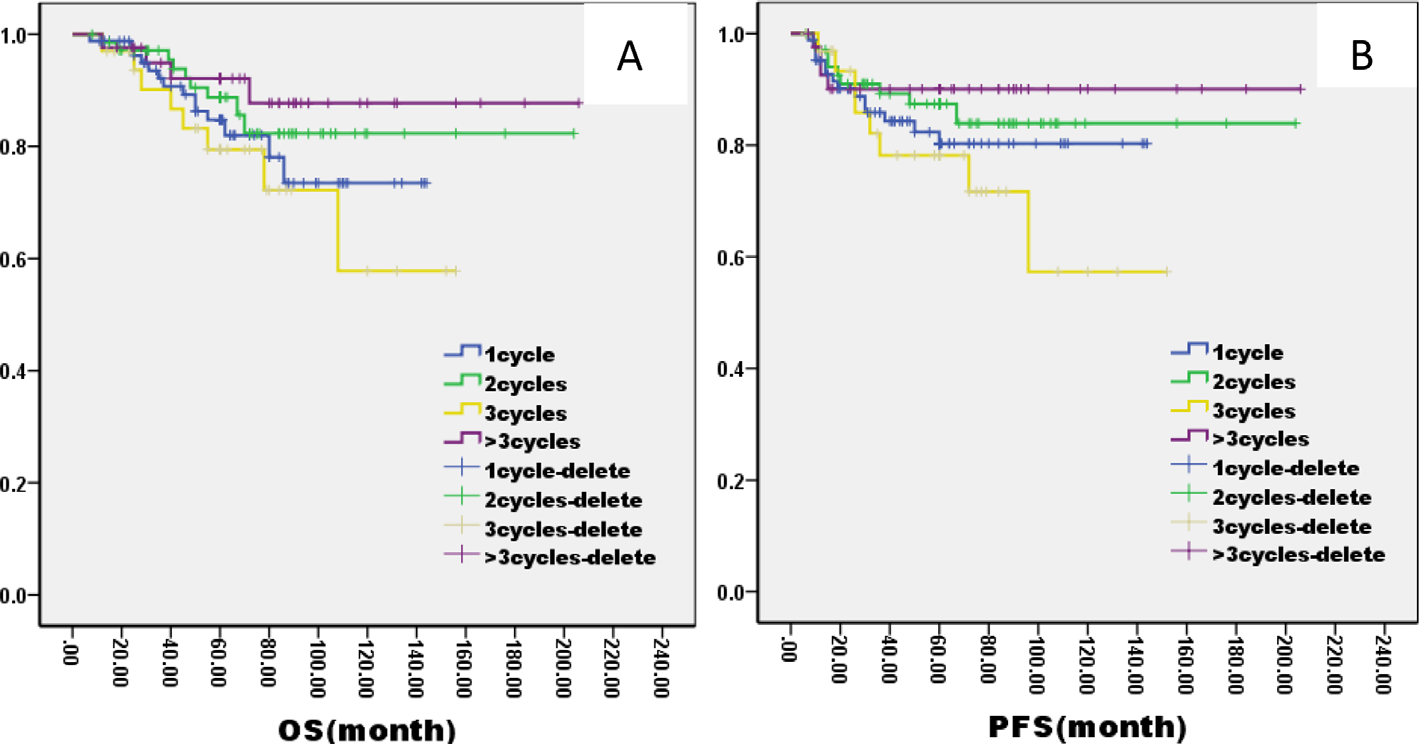
Kaplan–Meier curves of overall survival **(A)**, progression-free survival **(B)**, in LA-NPC patients treated with different AC cycles.

**Figure 7:**
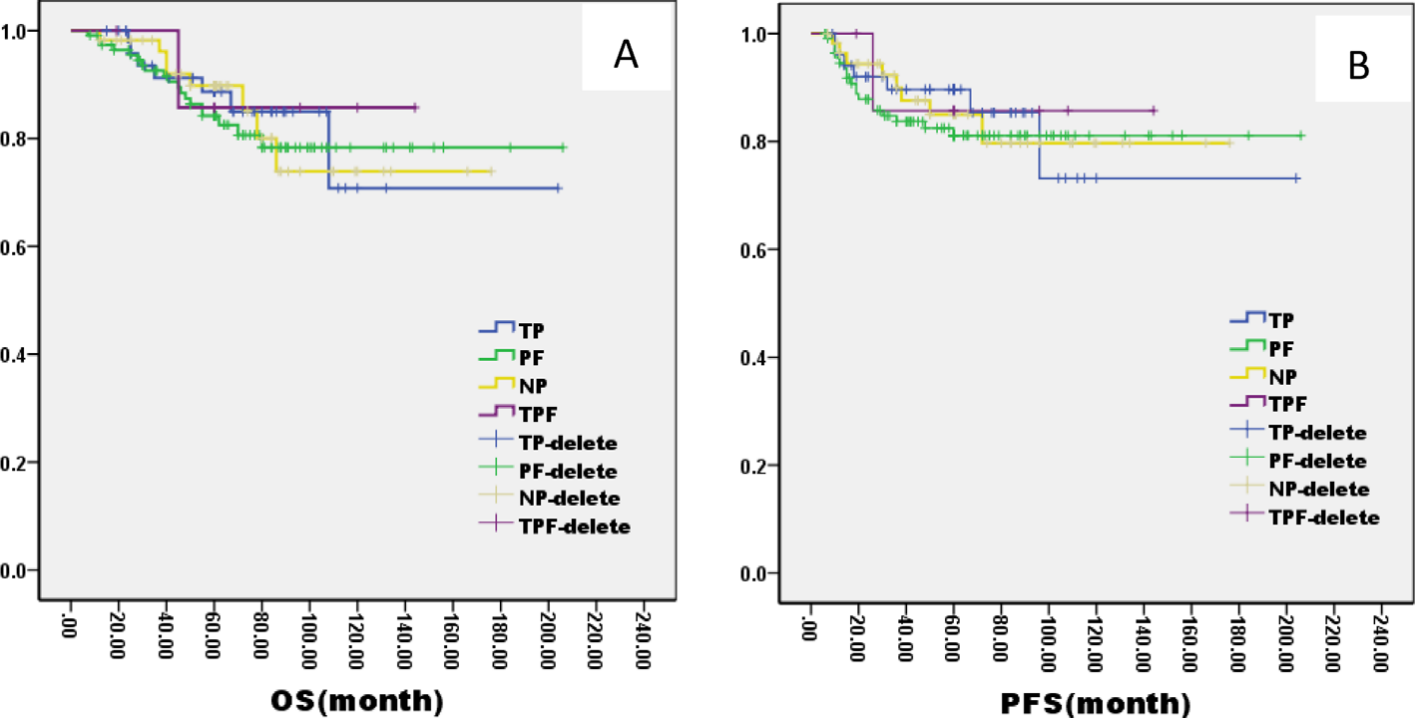
Kaplan–Meier curves of overall survival **(A)**, progression-free survival **(B)**, in LA-NPC patients treated with different chemotherapies.

The 5-year OS and PFS were significantly better in AC group than no AC group by the log-rank test in univariate analysis (OS: 82% vs. 72%, P=0.005; PFS: 81% vs. 69%, P=0.003, **Figure 5**). In the AC group, we analysis the effect of different chemotherapy cycles and chemotherapy regimens on survival time. In the AC group, there was no differential effect of chemotherapy cycles and chemotherapy regimens on patients’ OS and PFS (**Figures 6**, **7**).

### 3.3 Role of chemotherapy was different in different LA-NPC

**Figure 8:**
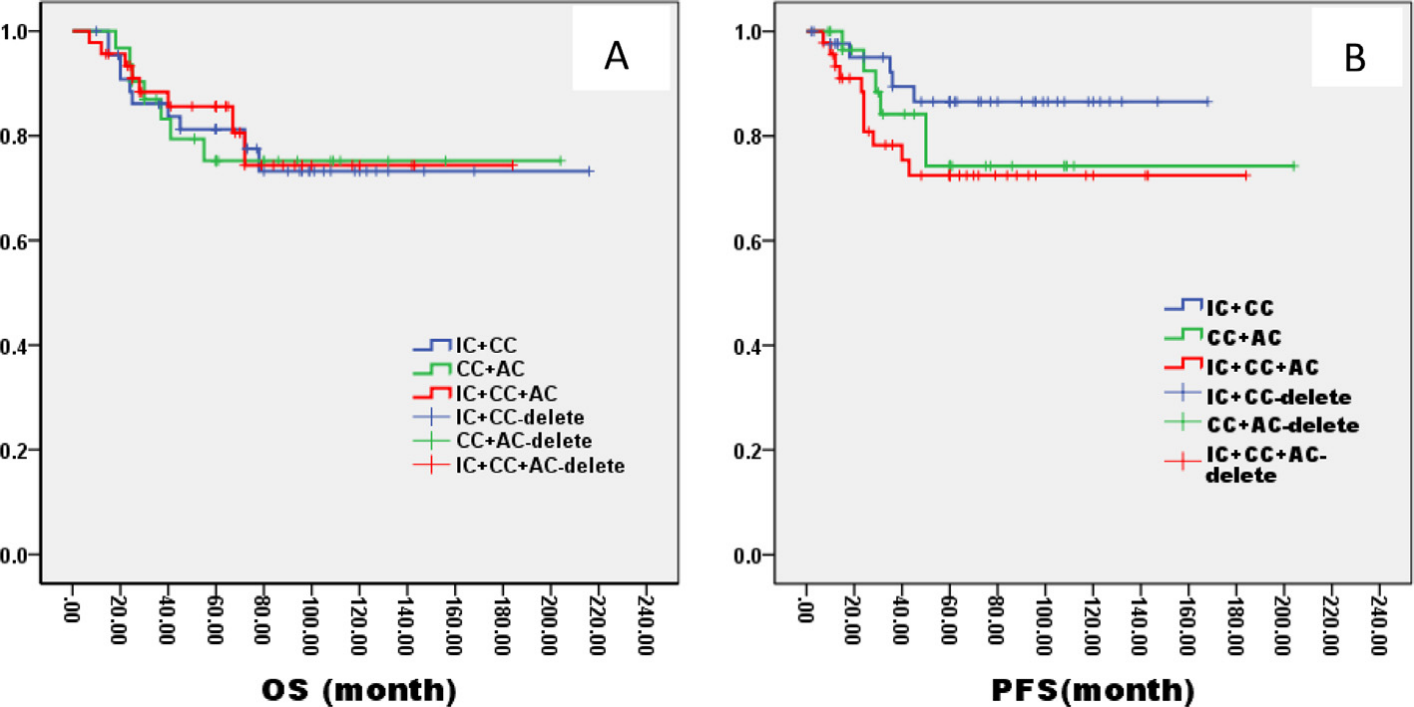
Kaplan–Meier curves of overall survival **(A)**, progression-free survival **(B)**, in LA-NPC patients staged in ThNl treated with different radiochemotherapy programmes. IC, Induction Chemotherapy (IC); AC, post-radiotherapy adjuvant chemotherapy; CC, concurrent chemoradiotherapy.

**Figure 9:**
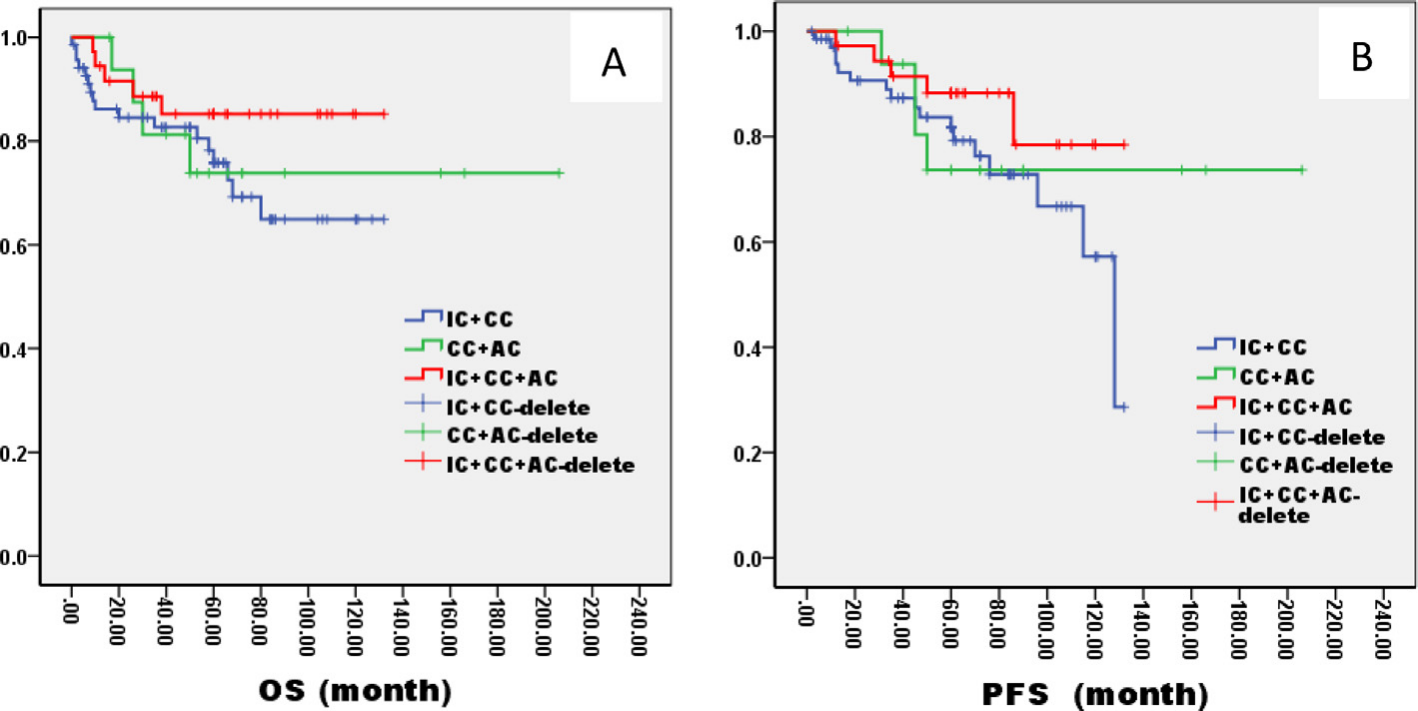
Kaplan–Meier curves of overall survival **(A)**, progression-free survival **(B)**, in LA-NPC patients staged in TlNh treated with different radiochemotherapy programmes. IC, Induction Chemotherapy (IC); AC, post-radiotherapy adjuvant chemotherapy; CC, concurrent chemoradiotherapy.

**Figure 10:**
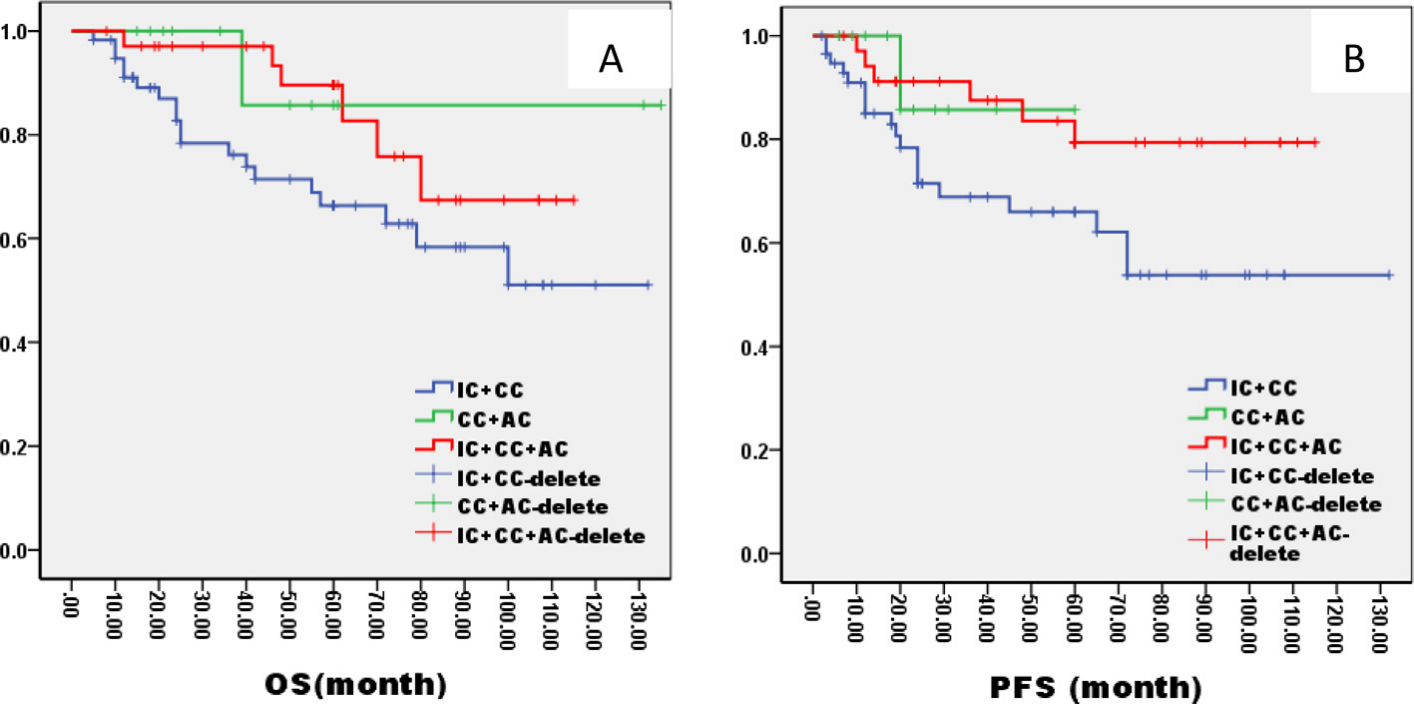
Kaplan–Meier curves of overall survival **(A)**, progression-free survival **(B)**, in LA-NPC patients staged in ThNh treated with different radiochemotherapy programmes. IC, Induction Chemotherapy (IC); AC, post-radiotherapy adjuvant chemotherapy; CC, concurrent chemoradiotherapy.

In the ThNl group, there was no significant difference among patients who received IC + CCRT, CCRT + AC, and IC + CCRT + AC (OS: 81% VS 75% VS 82%, P= 0.796; PFS: 78% VS 77% VS 79%, P= 0.685, **Figure 8**). In the TlNh group, there was no significant difference in OS and PFS among patients who received IC + CCRT, CCRT + AC, and IC + CCRT + AC (OS: 79% VS 74% VS 88%, P= 0.165; PFS: 70% VS 73% VS 85%, P= 0.154, **Figure 9**). In the ThNh group, OS and PFS were higher in patients receiving IC + CCRT + AC than in those receiving IC + CCRT, with no significant difference in the rest (OS: 85% VS 66% P= 0.013; PFS: 78% VS 62%, P= 0.049, **Figure 10**).

Multifactorial regression analysis was conducted to evaluate various therapeutic factors, including treatment modality (IC + CCRT vs IC + CCRT + AC vs CCRT + AC), IC regimens, IC cycles, AC regimens, AC cycles as potential independent prognostic factors for different TN group NPC. The analysis indicated that treatment modality, AC regimens were independent prognostic factors for OS and PFS of NPC in ThNh group (P<0.05), and while IC cycles was also independent prognostic factor for PFS of NPC in ThNh group (P<0.05). However, alcohol intake and the cumulative amount of drinking emerged as independent prognostic factors for NPC (P=0.046, 0.043) (**Supplementary Table S4**). There was no significant difference in other two TN groups.

### 3.4 Treatment toxicity

The most common adverse events (AEs) included mucositis, xerostomia, anemia, dermatitis, leukopenia, neutropenia, nausea, vomiting, and hepatotoxicity. There were no significant differences in the incidence of grade 3-4 AEs among the three groups. However, compared to patients treated with IC+CCRT or CCRT+AC, those treated with IC+CCRT+AC had a significantly higher incidence of grade 3 to 4 leukopenia (p=0.003) and neutropenia (p=0.002). The details are presented in **Supplementary Table S5**.

## 4 Discussion

NPC is notorious for its high metastatic potential, affecting up to one-third of patients in the highest-risk subgroups. Despite advancements in radiotherapy, NPC management remains challenging, with 15%–30% experiencing failure after radical treatment and 5% presenting with distant metastasis (9). For LA-NPC patients, IC + CCRT and CCRT +AC are both recommended treatment options. Additional chemotherapy paired with CCRT is regarded as a good therapeutic option for patients with LANPC. However, it is unclear whether additional chemotherapy should be given to these patients before or after concurrent systemic therapy/RT.

There is evidence supporting the use of induction chemotherapy followed by concurrent systemic therapy/RT for treatment of locoregionally advanced nasopharyngeal cancer. Two randomized phase III trials showed a survival benefit for induction chemotherapy followed by concurrent systemic therapy/RT, compared to concurrent systemic therapy/RT alone (10, 11). Results from multiple systematic reviews suggest that IC prior to systemic therapy/RT in patients with LA-NPC may potentially impact tumor control, compared to systemic therapy/RT without additional chemotherapy (12, 13). IC is considered a more practical approach: the target area of RT can be reduced by IC, subclinical metastases can be removed, and tumor lesions can be decreased. In addition, because it is carried out before CCRT, the general condition of patients is better, and they can tolerate the chemotherapy better (14). However, conflicting results exist, with one systematic review indicating no superior survival outcomes for IC preceding systemic therapy/RT than systemic therapy/RT alone or systemic therapy/RT +AC (15).

To some extent, our findings was consistent with the results of previous retrospective studies, not only did it show the 90-month OS and PFS in IC+CCRT group were significantly better only CCRT group, but also no more than 3 cycles IC was superior to more 3cycles in regards of OS, although there was no significant difference in 5-year OS and PFS between IC group and no IC group. This corresponds with a retrospective analysis proposing that two cycles of IC suffice, with additional cycles not confering extra survival benefits (16). Another retrospective analysis of 498 patients undergoing IC + CCRT revealed that after two IC cycles, a substantial proportion of patients achieved complete or partial tumor response (CR or PR). Additionally, a three-cycle IC regimen demonstrated improved OS and PFS among N2-3 patients with CR or PR, whereas it failed to benefit N0-1 or stable disease/disease progression patients (17). These findings indicated that it may be unnecessary to provide more than 3 cycles IC for LA-NPC patients.

Determining the optimal IC regimen (TPF, TP, PF, NP) remains crucial. Short-term efficacy comparisons reveal similarities between NP and TP groups, with no statistically significant differences observed in 3-year OS, DFS, locoregional recurrence free survival, or distant metastasis-free survival rates (18). A meta-analysis showed that certain cisplatin-based neoadjuvant chemotherapy regimens improved the prognosis of NPC and reduced the toxicity of CCRT. However, in view of survival rate and response rate, the best neoadjuvant chemotherapy regimen is not entirely consistent (19). Another meta-analysis emphasized the potential superiority of GP in enhancing survival outcomes for LA-NPC patients (20, 21). Others found that TPF has the highest probability to be the optimal IC regimen in LA-NPC than PF and TP. However, TPF induced worse AE, especially in ≥ grade 3 hematological toxicity and oral mucositis (22). Additionally, our study identified NP regimen as superior to TP, DF, and TPF regimens in terms of OS and PFS. However, comparative research on AE associated with different chemotherapy regimens remains limited.

Patients with LA-NPC face an elevated risk of disease recurrence, even after achieving complete clinical remission through standard-of-care treatments such as definitive CCRT, with or without IC. Consequently, the role of AC in NPC remains a contentious topic, emphasizing the imperative need for more efficacious adjuvant treatment modalities. Clinical trials and meta-analyses have both reported that CCRT +AC did not significantly improve the survival of patients with stage III–IVB NPC and could even increase the incidence of G3/4 toxicities (23, 24).

A study conducted from January 25, 2017, to October 25, 2018, enrolled 675 patients, with 406 receiving metronomic capecitabine and 202 receiving standard therapy. The 3-year failure-free survival rate was notably higher in the metronomic capecitabine group, further underscoring the potential benefits of AC (25). The trial 0099, which randomly assigned patients to external beam radiotherapy (EBRT) with concurrent cisplatin plus AC with PF for three cycles versus EBRT alone, the addition of chemotherapy also decreased local, regional, and distant recurrence rates (26). However, subsequent phase III randomized trials in Asia confirmed that CCRT without adjuvant PF similarly increased survival in endemic-area populations when compared with RT alone (27). The largest phase III randomized trials ever conducted in NPC comparing concurrent cisplatin/RT with (or without) adjuvant PF showed that adjuvant chemotherapy did not significantly improve survival following chemoradiation therapy (28). In our study, the 5-year OS and PFS demonstrated significant improvements in the AC group compared to the non-AC group, affirming the positive impact of AC on OS and PFS.

Tao et al (29) described the study in which 839 newly diagnosed LA-NPC patients were involved, and 443 receiving IC + CCRT while 396 undergoing IC + CCRT + AC. Notably, the inclusion of AC after IC + CCRT led to reduced distant metastases and superior OS and DFS outcomes compared to IC + CCRT. Our study categorized LA-NPC patients based on different T and N stages and explored various treatment strategies. In the ThNh group, patients who receiving IC + CCRT + AC exhibited higher OS and PFS compared to those receiving IC + CCRT, with no significant differences observed in other groups. The multifactorial analysis highlighted T-stage as a potential prognostic factor for OS, while T-stage and neck lymph node necrosis were identified as independent predictors of PFS and DMFS (30). The combination of IC, IMRT, and AC yielded favorable long-term survival outcomes for N3 disease patients, with neck lymph node necrosis and late T-stage serving as prognostic indicators of poorer outcomes (31). In summary, precision therapy stratified by T and N stages presents a promising avenue for optimizing the treatment of locally advanced nasopharyngeal carcinoma.

Several limitations of our study warrant mention. First, it was a retrospective study from a single treatment center, inevitably with internal bias. Second, some common factors affecting prognosis, such as EBV-DNA, were not included in the research because of the large amount of missing data. Third, although we eliminated selection bias, survival outcomes might be affected by other confounding factors. These limitations can be overcome by further prospective studies that include long-term results.

## 5 Conclusions

In summary, according to our retrospective study, CCRT combined with IC or AC seems to improve the 5-year OS and PFS of LA-NPC patients. The IC+CCRT +AC regimen seems to be the most beneficial approach for NPC patients with later T and N stages.
