# Supplementary material for: Long-term outcomes of induction chemotherapy followed by concurrent chemoradiotherapy and adjuvant chemotherapy for locoregionally advanced nasopharyngeal carcinoma: a retrospective study
Source: Front Oncol. 2024 Nov 27;14:1475176. doi: 10.3389/fonc.2024.1475176 (PMC11632233; doi:10.3389/fonc.2024.1475176)
Supplement: Supplementary file 1 [file Table1.docx]

**Table 1 Groups of NPC**

|  | **ThNl** | **TlNh** | **ThNh** |
| --- | --- | --- | --- |
| **T stage** | 3-4 | 0-2 | 3-4 |
| **N stage** | 0-1 | 2-3 | 2-3 |

**Table 2 Treatment characteristics**

| **treatment type** | ***N*** | **Percentage** |
| --- | --- | --- |
| **Chemotherapy** |  |  |
| induction | 173 | - |
| concurrent | 352 | - |
| adjuvant | 179 | - |
| **induction chemotherapy agent** | | |
| TP | 43 | 24.86% |
| DF | 73 | 42.20% |
| NP | 41 | 23.70% |
| TPF | 10 | 5.78% |
| Others | 6 | 3.46% |
| **adjuvant chemotherapy agent** | | |
| TP | 39 | 21.79% |
| DF | 88 | 49.16% |
| NP | 45 | 25.14% |
| TPF | 5 | 2.79% |
| Others | 2 | 1.12% |

**Table 3 Baseline characteristics of NPC patients in different treatment groups**

|  | IC＋CCRT | CCRT+AC | IC+CCRT+AC | *P* |
| --- | --- | --- | --- | --- |
| Age | | | | |
| ≤50 | 103 | 39 | 80 | 0.227 |
| ＞50 | 70 | 21 | 39 |  |
| Sex | | | | |
| male | 132 | 46 | 93 | 0.817 |
| female | 41 | 14 | 26 |  |
| TN | | | | |
| ThNl | 46 | 31 | 46 | 0.000 |
| TlNh | 69 | 17 | 38 |  |
| ThNh | 58 | 12 | 35 |  |
| Smoke | | | | |
| Y | 44 | 12 | 37 | 0.321 |
| N | 130 | 47 | 82 |  |
| Drink | | | | |
| Y | 87 | 26 | 61 | 0.848 |
| N | 87 | 33 | 58 |  |

**Table 4 Results of multivariate analysis for LA-NPC**

| Group | Endpoints | variable | HR | *P* |
| --- | --- | --- | --- | --- |
| ThNh | os | Treatment modality | 0.57(0.27-1.22) | ***0.04*** |
|  |  | IC regimens | 1.64(1.04-2.61) | ***0.03*** |
|  |  | IC cycles | 0.78(0.49-1.23) | 0.28 |
|  |  | AC regimens | 1.06(0.56-2.00) | 0.86 |
|  |  | AC cycles | 1.57(0.86-2.86) | 0.14 |
|  | PFS | Treatment modality | 0.57(0.27-1.12) | ***0.03*** |
|  |  | IC regimens | 1.61(1.00-2.59) | ***0.05*** |
|  |  | IC cycles | 0.73(0.46-1.15) | ***0.05*** |
|  |  | AC regimens | 1.04(0.55-1.96) | 0.90 |
|  |  | AC cycles | 1.6(0.89-2.89) | 0.12 |
| ThNl | os | Treatment modality | 1.68(0.73-3.87) | 0.22 |
|  |  | IC regimens | 1.45(0.87-2.42) | 0.16 |
|  |  | IC cycles | 0.71(0.42-1.18) | 0.18 |
|  |  | AC regimens | 1.15(0.79-1.67) | 0.48 |
|  |  | AC cycles | 0.88(0.47-1.66) | 0.69 |
|  | PFS | Treatment modality | 1.58(0.68-3.68) | 0.29 |
|  |  | IC regimens | 1.43(0.86-2.39) | 0.17 |
|  |  | IC cycles | 0.71(0.43-1.19) | 0.19 |
|  |  | AC regimens | 1.16(0.79-1.69) | 0.43 |
|  |  | AC cycles | 0.92(0.49-1.73) | 0.8 |
| TlNh | os | Treatment modality | 1.03(0.31-3.48) | 0.96 |
|  |  | IC regimens | 0.88(0.49-1.59) | 0.67 |
|  |  | IC cycles | 0.69(0.39-1.21) | 0.19 |
|  |  | AC regimens | 0.85(0.38-1.92) | 0.69 |
|  |  | AC cycles | 0.82(0.45-1.49) | 0.51 |
|  | PFS | Treatment modality | 0.98(0.29-3.36) | 0.97 |
|  |  | IC regimens | 0.92(0.51-1.67) | 0.78 |
|  |  | IC cycles | 0.68(0.39-1.20) | 0.18 |
|  |  | AC regimens | 0.87(0.39-1.98) | 0.75 |
|  |  | AC cycles | 0.81(0.45-1.47) | 0.49 |

**Table 5 The treatment toxicities of patients with LA-NPC**

| Toxic reactions (3-4) | IC＋CCRT | CCRT+AC | IC＋CCRT+AC | *P* |
| --- | --- | --- | --- | --- |
| mucositis | 34(19.7%) | 12(20.0%) | 25(21.0%) | 0.879 |
| Xerostomia | 5(2.9%) | 2(3.33%) | 4(3.36%) | 0.932 |
| Anemia | 9(5.2%) | 3(5.0%) | 7(5.88%) | 0.989 |
| dermatitis | 8(4.62%) | 3(5.0%) | 6(5.04%) | 0.095 |
| Leukopenia | 10(5.78%) | 6(10%) | 21(17.65%) | **0.003** |
| neutropenia | 13(7.51%) | 5(8.33%) | 23(19.33%) | **0.002** |
| nausea | 21(12.14%) | 8(13.33%) | 16(13.45%) | 0.835 |
| vomiting | 16(9.25%) | 7(11.67%) | 14(11.76%) | 0.096 |
| hepatoxicity | 6(3.47%) | 2(3.33%) | 5(4.20%) | 0.085 |
